# Obsessive-compulsive disorder: does CBT with exposure and response (ERP) prevention work?

**DOI:** 10.1192/bjo.2021.757

**Published:** 2021-06-18

**Authors:** Jemma Reid, Naomi A Fineberg, Lynne Drummond, Keith Laws, Matteo Vismara, Benedetta Grancini, Davis Mpavaenda

**Affiliations:** 1 Hertfordshire Partnership University NHS Foundation Trust; 2 South West London and St George's Mental Health NHS Trust; 3 University of Hertfordshire; 4 University of Milan

## Abstract

**Aims:**

Since the 1970s treatment for obsessive Compulsive Disorder (OCD) has consisted of the the application of drugs acting on the serotonin system of the brain or psychological treatments using graded exposure. Although there is a large number of studies on psychological treatments, they often are underpowered. Other major methodological issues include ignoring the effects of medication during the trial, using a variety of techniques and using waiting list data as controls.

We decided to systematically review and perform a meta-analysis on randomised controlled trials (RCTs) of CBT with ERP (abbreviated to ERP)1.

**Method:**

The study was preregistered in PROSPERO (CRD42019122311). RCTs incorporating ERP were examined. The primary outcome was the end-of-trial symptoms scores for OCD. In addition, factors which may have influenced the outcome including patient-related factors, type of control intervention, researcher allegiance and other potential forms of bias were examined. The moderating effects of patient-related and study-related factors including type of control intervention and risk of bias were also examined.

**Result:**

Overall, 36 studies were included in the analyses, involving 537 children/adolescents and 1483 adults (total 2020 subjects). A total of 1005 received ERP and the remainder a variety of control treatments. Initial results showed that ERP had a large effect size compared with placebo treatments. This was more marked in younger than older persons. However, whereas ERP was markedly more effective than waiting list or psychological control, this positive effect size disappeared when it was compared with other psychological treatments.

When ERP was compared against psychopharmacological treatment it initially appeared significantly superior but this reduced to marginal benefit when compared with adequate doses of appropriate medication.

The majority of studies were performed where there may be expected to be researcher allegiance to ERP and in these studies the effect size was large. In contrast, in the 8 studies considered to have low risk of researcher bias, ERP was found to be ineffective.

**Conclusion:**

Although on initial sight CBT incorporating ERP seems to be highly efficacious in the treatment of OCD, further analysis revealed that this varied depending on the choice of comparator control. In addition there are considerable concerns about methodological rigour and reporting of studies using CBT with ERP. Further studies examining the role of researcher bias and allegiance are needed.

Ref : 1 Jemma E Reid, Keith R Laws, Lynne Drummond, Matteo Vismara, Benedetta Grancini , Davis Mpavaenda, Naomi A Fineberg (2021) Cognitive Behavioural Therapy with Exposure and Response Prevention in the treatment of Obsessive-Compulsive Disorder: A systematic review and meta-analysis of randomised controlled trials. Comprehensive Psychiatry , in press.

